# The Hidden Pain: Understanding Smartphone Pinky Awareness and Risk Perception in the Eastern Province Population

**DOI:** 10.7759/cureus.64350

**Published:** 2024-07-11

**Authors:** Saud N Aldanyowi, Lynah M Al Haboob, Nora I Al Mssallem

**Affiliations:** 1 Orthopedics, King Faisal University, Al Hofuf, SAU; 2 Medicine, King Faisal University, Al Hofuf, SAU; 3 Surgery, King Faisal University, Al Hofuf, SAU

**Keywords:** saudi arabia, eastern province population, pinky, smartphones, awareness, orthopedic

## Abstract

Background/objective: The detrimental effects of smartphone addiction impair hand functions and pinch strength. One prominent issue is the occurrence of “smartphone pinky,” which leads to dysfunction and pain in the fifth finger. This study aims to assess the level of awareness regarding smartphone pinky and its associated risk factors among the population of the Eastern Provinces.

Methods: This cross-sectional study was carried out on 500 participants from the Eastern Province of Saudi Arabia. Participants voluntarily took part in the research, which spanned from July 2023 to February 2024. Data collection was carried out using a semi-structured questionnaire designed to gauge awareness of smartphone pinky and its contributing factors within the Eastern Province population.

Results: The study included 500 participants. This study showed that about half of the participants (48.8%) use smartphones for five to eight hours during the day. Moreover, about two-thirds of them (64.6%) held the smartphone in the wrong way. The majority of participants 74.4% have not heard about the smartphone pinky. Also, the results reveal that less than half of the participants (45.8%) think that the smartphone pinky can affect daily life.

Conclusions: This study concluded that the majority of participants have not heard about smartphone pinky and do not have awareness of the risk factors of smartphone use.

## Introduction

Smartphone usage has become increasingly prevalent in modern society, with a myriad of medical conditions linked to its excessive use [1]. There exists a significant biomechanical hazard, particularly concerning the neck, wrists, and thumbs, attributed to recurring muscle tension, habitual movements, and poor posture [2].

The rise in smartphone usage has been correlated with an elevated incidence of musculoskeletal pain [3]. During smartphone use, individuals often maintain static positions for extended durations, leading to muscle fatigue and heightening the likelihood of musculoskeletal disorders.

Consequently, the functionality of the hands and pinch strength may be adversely affected by smartphones. Moreover, many smartphone users report experiencing wrist or finger discomfort. This discomfort may be associated with specific types of pinky injuries and inflammation of the tendon sheath in the extensor pollicis brevis, abductor pollicis longus, and phalanx of the finger [4]. The condition known as “smartphone pinky” typically impacts the middle phalanx of the fifth finger and can result in pain and dysfunction along the same finger [5]. However, smartphone usage has been linked to various social and physical issues that affect several limbs, particularly the little finger [6].

A recent study conducted in India revealed that students who used their smartphones for prolonged periods reported neck discomfort in 46.9% of cases and thumb pain in 29.2%. In contrast, 66.4% of participants in another study were identified as smartphone addicts, and 19.1% tested positive for the Finkelstein test [7]. In yet another study, 79% of participants aged between 18 and 44 reported using their smartphones almost constantly throughout the day, with only a two-hour break. This phenomenon is referred to as non-chemical or technical addiction [8].

Unfortunately, there is not much data on the effects of widespread smartphone use. There is limited evidence to suggest that prolonged use of cell phones may be harmful to fingers, especially the pinky [9]. The Eastern Province population's knowledge of smartphone pinky and its risk factors was also assessed in this cross-sectional study. Our hypothesis was that there would be a notable variation in the fifth finger's knowledge of smartphones and their associated risks.

## Materials and methods

Study design

The study design involves a cross-sectional approach, encompassing 500 participants over an eight-month duration from July 2023 to February 2024. Selection criteria for inclusion were both male and female individuals aged 16 years or older, holding Saudi citizenship, and demonstrating willingness to participate in the study by providing informed written consent. Exclusion criteria comprised non-Saudi nationality, any known medical conditions leading to upper limb and hand pain, recent fractures in the hand and upper limb, traumatic injuries within the past six months, congenital abnormalities, and severe surgical and neurological disorders.

Sample size estimation

The subject's size was calculated by utilizing the formula for estimating proportion: n = Zα2 P (1 − P)/d2, where Zα = 1.96; P = 75.8%; from the previously published article and d = 5%. The estimated sample size was a minimum of 500 participants.

Data collection and survey development

One tool used in this study for data collection is a semi-structured questionnaire that consists of questions related to sociodemographic characteristics, previous health history as well as smartphone usage habits.

Demographic variables

Sociodemographic characteristics include age, sex, residence, and occupation. Previous health history includes history of chronic diseases or any bone disorders. Smartphone usage-related data were computed from the previous validated questionnaires.

Survey validation and administration

The survey questionnaire draft was not subjected to content validation as it is an adopted tool. The final version of the questionnaire was initially tested on a sample of five representative individuals from the population to assess response time, which did not exceed 20 minutes for completion.

Statistical analysis

To assess the reliability of the questionnaire, Cronbach's alpha was calculated. The data were analyzed utilizing IBM Statistical Package for Social Science (SPSS) Statistics for Windows version 20.0 (IBM Corp., Armonk, NY) and MedCalc software version 15.8.0 (Medcal, Lima, Peru). Quantitative data were presented as a median and interquartile range, while qualitative data were presented as numbers and percentages. The normality of the data was assessed using the Shapiro-Wilk test. For normally distributed data, the independent samples t-test was applied. Non-normally distributed data were analyzed using the nonparametric Mann-Whitney U test. Comparison of qualitative variables was performed using the Chi-square (χ2) test and Fisher's Exact Test, as appropriate.

## Results

This cross-sectional study was performed on 500 participants. Among the participants, 105 were male and 395 were female. Table 1 reveals that two-thirds of participants (63.6%) were between the ages of 16-25 years.

**Table 1:** Demographic data of the studied participants.

		n=500
Age (years)	16-25	318 (63.6%)
	26-35	34 (6.8%)
	36-45	74 (14.8%)
	46-55	55 (11%)
	56-60	19 (3.8%)
Sex	Male	105 (21%)
	Female	395 (79%)

**Figure 1:**
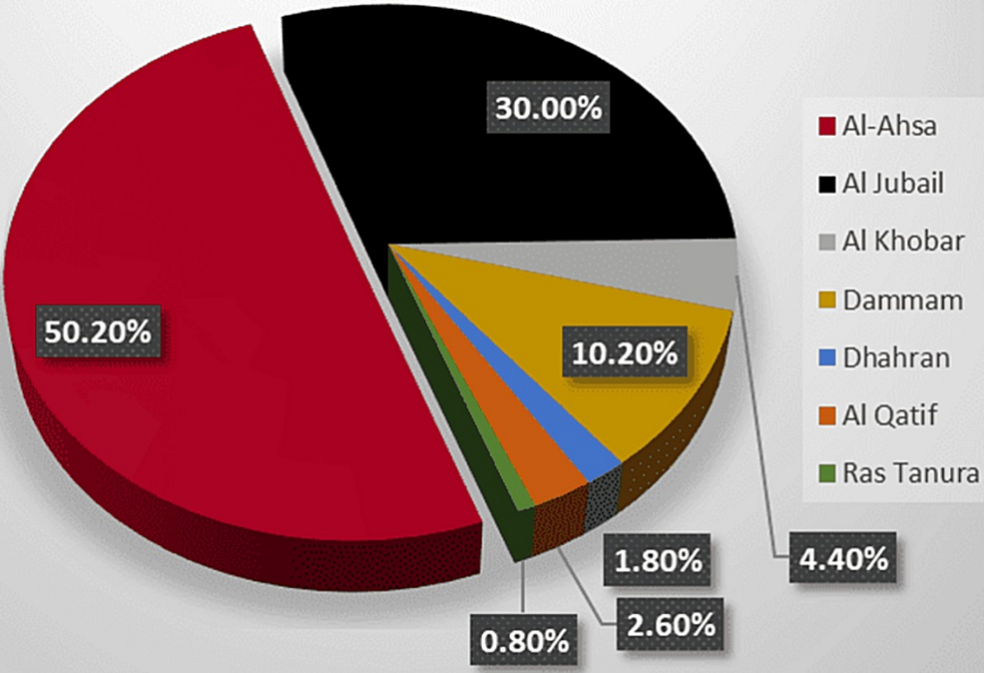
Residence of the studied participants.

Figure 1 shows the residence of the studied participants. About half of the participants (50.2%) were from Al-Ahsa. While about one-third of them (30%) were from Al Jubail.

**Figure 2:**
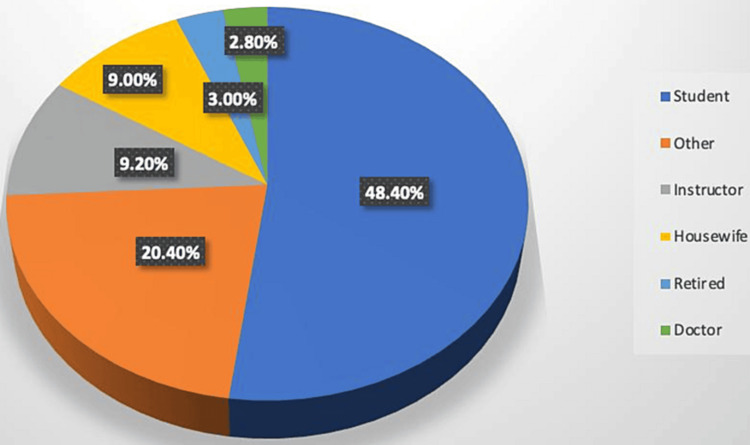
Occupation of the studied participants. Category Other includes (but is not limited to) accountants, customer service specialists, pilots, engineers, etc.

Figure 2 represents in relation to occupation. The highest proportion of participants (48.4%) were students, while the lowest proportion of them (2.8%) were doctors.

Table 2 indicates the history of the studied participants where a small proportion (14.2%) suffered from chronic diseases, and slightly more than one-third (33.4%) of participants had bone problems affecting adjacent joints or the neck.

**Table 2:** History of the studied participants.

		n=500
Do you suffer from any chronic diseases such as: DM, HTN or rheumatism?	Yes	71 (14.2%)
	No	429 (85.8%)
Do you suffer from any bone problems: (neck, shoulder, elbows, or wrist)?	Yes	167 (33.4%)
	No	333 (66.6%)

Table 3 mentions smartphone usage-related data of the studied participants. The majority (79.6%) of participants used right hand to hold the smartphones while less than one-quarter of them (20.4%) used left hand.

**Table 3:** Smartphone usage-related data of the studied participants.

		n=500
Which hand do you use to hold the smartphone?	Right	398 (79.6%)
	Left	102 (20.4%)
How big is the phone you use compared to your hand?	Suitable	370 (74%)
	Large	111 (22.2%)
	Small	19 (3.8%)
How many hours do you use your smartphone during the day?	Less than 1 hour	5 (1%)
	2-4 hours	110 (22%)
	5-8 hours	244 (48.8%)
	More than 10 hours	141 (28.2%)
In your opinion, what is the correct way to hold a smartphone?	The correct way	380 (76%)
	The wrong way	120 (24%)
How often do you hold your smartphone?	The correct way	177 (35.4%)
	The wrong way	323 (64.6%)

Furthermore, about half of participants (48.8%) use smartphone from five to eight hours during the day. Moreover, about two-third of them (64.6%) held the smartphone in the wrong way. Table 4 displays that the majority of participants (74.4%) have not heard about smartphone pinky and 60.16% of them reported that they obtained their knowledge through social media. Also, regarding the time of smartphone use during the day, 62.8% of participants reports more than 10 hours of usage. Also, the results reveal that less than half of participants (45.8%) think that smartphone pinky can affect daily life.

**Table 4:** Smartphone usage-related data of the studied participants.

		n=500
Have you heard about the smartphone pinky?	Yes	128 (25.6%)
	No	372 (74.4%)
Where did you hear about the smartphone pinky?	Social media	77 (60.16%)
	Awareness campaigns	10 (7.81%)
	Newspaper	2 (1.56%)
	Other	39 (30.47%)
What do you think are the symptoms of a smartphone pinky?	Hand pain	115 (23%)
	Muscles weakness	19 (3.8%)
	Bone deformities	64 (12.8%)
	All of above	302 (60.4%)
Are you suffering from any of these symptoms?	Yes	161 (32.2%)
	No	339 (67.8%)
Do you have a history of problems or injury to your pinky finger?	Yes	34 (6.8%)
	No	466 (93.2%)
Do you think the smartphone pinky is a serious condition?	Yes	95 (19%)
	No	75 (15%)
	Perhaps	330 (66%)
Have you been suffering from smartphone pinky?	Yes	78 (15.6%)
	No	422 (84.4%)
How much time of smartphone use during the day do you think could cause smartphone pinky?	Less than 1 hour	18 (3.6%)
	2-3 hours	48 (9.6%)
	5-8 hours	120 (24%)
	More than 10 hours	314 (62.8%)
Do you think a smartphone pinky can affect daily life?	Yes	229 (45.8%)
	No	58 (11.6%)
	Perhaps	213 (42.6%)

## Discussion

A smartphone is a portable device equipped with a specially designed computerized system that allows instant access to the Internet and facilitates sending and receiving emails. The global usage of smartphones has been consistently rising over time [10]. Smartphones are known to have a number of dangers to a user's physical, psychological, and social well-being despite having a touchscreen that makes for easy one-button interaction by merging input and output into one interface [4]. On the fifth (little) finger, the majority of these hazards result in pain and inflammation. Therefore, the likelihood of smartphone pinky will decrease by using hand-and-finger awareness to input across the entire surface of the device. Thus, it is essential to comprehend the behavior and ergonomics of each finger when holding and using a smartphone [11].

Using smartphones for extended periods can lead to discomfort and potential injury, particularly to the fingers and wrists. To minimize these risks, it is important to adopt healthy habits and ergonomic practices when holding your smartphone. The following are some tips.

1: Whenever possible, use both hands to hold and operate your smartphone to distribute the load and reduce strain on any single finger.

2: Avoid holding your smartphone for prolonged periods by taking breaks to stretch and rest your fingers.

3: Additionally, using a phone stand or holder can help you maintain a neutral wrist position and reduce the need to grip the phone tightly.

4: Avoid cradling the phone between your neck and shoulder as this position can cause strain not only to your fingers but also to your neck and shoulders.

5: Lastly, utilize voice commands and shortcuts to perform tasks and reduce the need for excessive tapping.

Research has shown that improper smartphone use can lead to musculoskeletal issues, including reduced hand-grip strength and pinch-grip strength, as well as increased ergonomic risks affecting posture and muscle use [12].

In terms of sex, the study's observations showed that women made up the bulk of the participants. This observation was consistent with Megna et al., who noted that women constituted the majority of study participants who used smartphones [13]. Particularly among young people, smartphone and other mobile device use has become commonplace. According to the researcher, younger people may not have been as aware of smartphone pinky as older participants. Furthermore, proportionally stated, the largest percentage of participants in our study were students. This observation aligns with Demirci et al., who found that all participants in their study, who were university students, habitually used smartphones with a single hand [14]. Regarding the smartphone usage patterns of the participants in our study, the observations indicate that most individuals utilized their right hand to hold the device. This observation corresponds with the findings of Megna et al., who highlighted that the majority of their study's participants were right-handed [13]. Furthermore, this study shows that about half of the participants use smartphones for five to eight hours during the day. The findings were in contradiction with Qasim et al., who mentioned that the majority of their respondents utilized their smartphones for a period of more than three hours each day [15].

Most participants in the study showed no signs of smartphone pinky. Currently, smartphones play a crucial role in the daily lives of students. Furthermore, the study found that fewer than half of the participants believed that smartphone pinky could impact their daily activities [16]. This is consistent with Duke et al., whose research also highlighted a significant negative impact of smartphone usage on the personal lives of participants [17].

The advantage of this study was its cost-effectiveness and efficiency in terms of time. However, a limitation was its geographic scope, being confined to the Eastern Province, which may not reflect the views of the entire country's population. Future studies could expand nationwide to gather more comprehensive data.

## Conclusions

In summary, this study highlights the prevalent lack of awareness regarding smartphone pinky and its associated risks among the population of the Eastern Province. Despite the widespread use of smartphones, particularly among younger individuals and students, many participants remain uninformed about the potential health impacts, including the risk of developing smartphone pinky. Our observations indicate that a significant proportion of participants use their smartphones for extended periods and in ergonomically incorrect ways, contributing to potential musculoskeletal issues.

The findings underscore the need for increased public education and awareness campaigns to inform users about the importance of proper smartphone handling techniques to prevent discomfort and long-term health problems. Additionally, future research should aim to include a broader geographic scope to provide more comprehensive data and further explore the implications of smartphone usage on hand health. Preventive measures and ergonomic guidelines should be promoted to mitigate the risks associated with excessive smartphone use, thereby enhancing the overall well-being of the population.
